# Governance and pharmacovigilance in Brazil: a scoping review

**DOI:** 10.1186/s40545-016-0053-y

**Published:** 2016-02-08

**Authors:** Kathy Moscou, Jillian C. Kohler, Anita MaGahan

**Affiliations:** Leslie Dan Faculty of Pharmacy, University of Toronto, 144 College Street, Toronto, Ontario M5S 3M2 Canada; Munk School of Global Affairs, 1 Devonshire Place (At Trinity College), Toronto, Ontario M5S 3K7 Canada; Rotman School of Management, Joseph L. Rotman School of Management, 105 St George Street, Toronto, Ontario M5S 3E6 Canada

**Keywords:** Brazil, Drug safety, Governance, Pharmacovigilance, Regulation pharmaceutical policy

## Abstract

**Background:**

This scoping review investigates the relationship between governance, pharmacovigilance, and Agencia Nacional de Vigilancia Sanitaria (ANVISA) in Brazil, which has authority over Brazil's national pharmaceutical policy, drug registration and coordination of the national pharmacovigilance system. The purpose is to investigate opportunities for effective pharmacovigilance.

**Methods:**

Sixty-three terms pertaining to pharmacovigilance in Brazil and ANVISA, global institutions, pharmaceutical industry, and civil society were searched in thirteen relevant databases on November 17-18, 2013. Using a pharmacogovernance framework we analyzed ANVISA's pharmacogovernance: the manner in which governing structures, policy instruments, and institutional authority are managed to promote societal interests for patient safety due to medication use. The integration of transnational policy ideas for regulatory governance into pharmacogovernance in Brazil was also investigated.

**Results:**

Brazil's policy, laws, and regulations support ANVISA's authority to ensure access to safe medicines and health products however ANVISA's broad mandate and gaps in pharmacogovernance account for regional disparities in monitoring and assessing drug safety. Gaps in pharmacogovernance include: equity and inclusiveness; stakeholder coordination; effectiveness and efficiency; responsiveness; and intelligence and information.

**Conclusions:**

Pharmacogovernance that addresses 1) regional resource disparities, 2) federal and state lack of coordination of pharmacovigilance regulations, 3) asymmetric representation in the pharmaceutical regulatory agenda and which 4) disaggregates regulatory authority over health and commercial sectors would strengthen pharmacovigilance in Brazil.

**Electronic supplementary material:**

The online version of this article (doi:10.1186/s40545-016-0053-y) contains supplementary material, which is available to authorized users.

## Introduction

Pursuant to Article 196 of the Brazilian Constitution, all Brazilians have the right to health [1–3]. The Constitutional commitment to health for its population includes access to safe, effective, quality essential medicines; guidelines to promote rational use; and cost control [3] as expressed in Brazil’s National Medicines Policy (NMP). One key challenge the Federal government has faced is how to determine what governance, regulations and policy instruments best fulfill Brazilian’s constitutional right to health; including assuring nationwide equity in monitoring, assessing, and communicating drug safety risk. Accordingly, we investigate pharmacogovernance in Brazil and the Agência Nacional de Vigilância Sanitária (ANVISA) regulatory governance for their impact on pharmacovigilance (the science and activities relating to detecting, assessing, understanding and preventing adverse effects or other possible drug-related problems).

We define ***pharmacogovernance*** as *the manner in which governing structures; policy instruments and institutional authority (ability to act, implement and enforce norms, policies and processes) are managed to promote societal interests for patient safety and protection from adverse drug events*. Pharmacogovernance embraces a culture that supports drug safety and contributes to maintaining a healthy population, which the state and corporate sector advances as aligned with pharmacovigilance [4]. The absence of strong pharmacogovernance undermines stewardship for postmarket drug safety, safety signal detection, risk communication and rational medicine use. Weak pharmacogovernance therefore entails a lack of oversight and accountability that may negatively affect pharmacovigilance by:

- Creating opportunities for corruption to emerge [5–7];
- Creating institutional conflicts of interest, whereby regulators are dually responsible for protecting patient safety and industry competitiveness [8–10];
- De-incentivizing adoption of legislation and norms for pharmacovigilance; and
- De-incentivizing detection of adverse drug reactions (ADR) [5–7].

These negative outcomes of deficient pharmacogovernance are at odds with public health needs and the constitutional right to health in Brazil.

We also investigated whether governance by the Agência Nacional de Vigilância Sanitária (ANVISA) and support from the international community are sufficient to ensure postmarket drug safety across Brazil. Specifically, we investigated how global actors’ policy ideas for regulatory governance (e.g., transparency and accountability) were integrated into pharmacogovernance in Brazil. Global actors were broadly defined as agents that influence public policy in multiple countries. They included employees of the World Health Organization/Pan American Health Organization (WHO/PAHO), US Food and Drug Administration (FDA), Global Fund, European Medicines Agency (EMA) and others that have provided pharmacovigilance guidelines, best practices, training, regulatory norms, technical expertise and access to global knowledge networks [11–16].

We employ *Ideation Theory* to frame our understanding of how and why global actors’ policy ideas and norms pertaining to pharmacovigilance have gained traction in Brazil. *Ideation Theory* suggests that a meaningful feature of global actors is their capacity to convert ‘soft power’ into ‘hard power’ whereby global actors’ policy ideas and knowledge influence the policy agendas, policy tools, legislation, and practices of recipient countries [17]. Soft power represents a persuasive approach that is taken to shape or co-opt government policy preferences or public opinion. The power to influence rests in perceived legitimacy or shared values [18]. Norms are presented as a ‘toolbox’ from which countries choose according to perceived relevance.

Ideation Theory suggests that policy uptake usually requires collaboration between national and transnational policy actors’ with technical and financial support [19]. New ideas (e.g., such as the use of policy tools for analyses of regulatory policy) are adopted to the extent that they respond to concrete policy problems, resonate with the interest and ideas of key actors and are brought to the attention of relevant public agencies that have the structural capacity to implement the new ideas [20]. The policy ideas are reinforced through peer learning. Peer learning is used as a strategy for the diffusion of global development agencies’ policy ideas to poor and developing countries [17, 21].

Our paper is organized as follows. First, the evolution of pharmacovigilance and regulatory authority over postmarket drug safety in Brazil is described. Next, our search methodology is described following the STARLITE reporting criteria. Following, our research findings are reported for each of the literature typologies we identified. Lastly, recommendations to advance pharmacogovernance and pharmacovigilance in Brazil are provided.

## Background

**Fig. 1:**
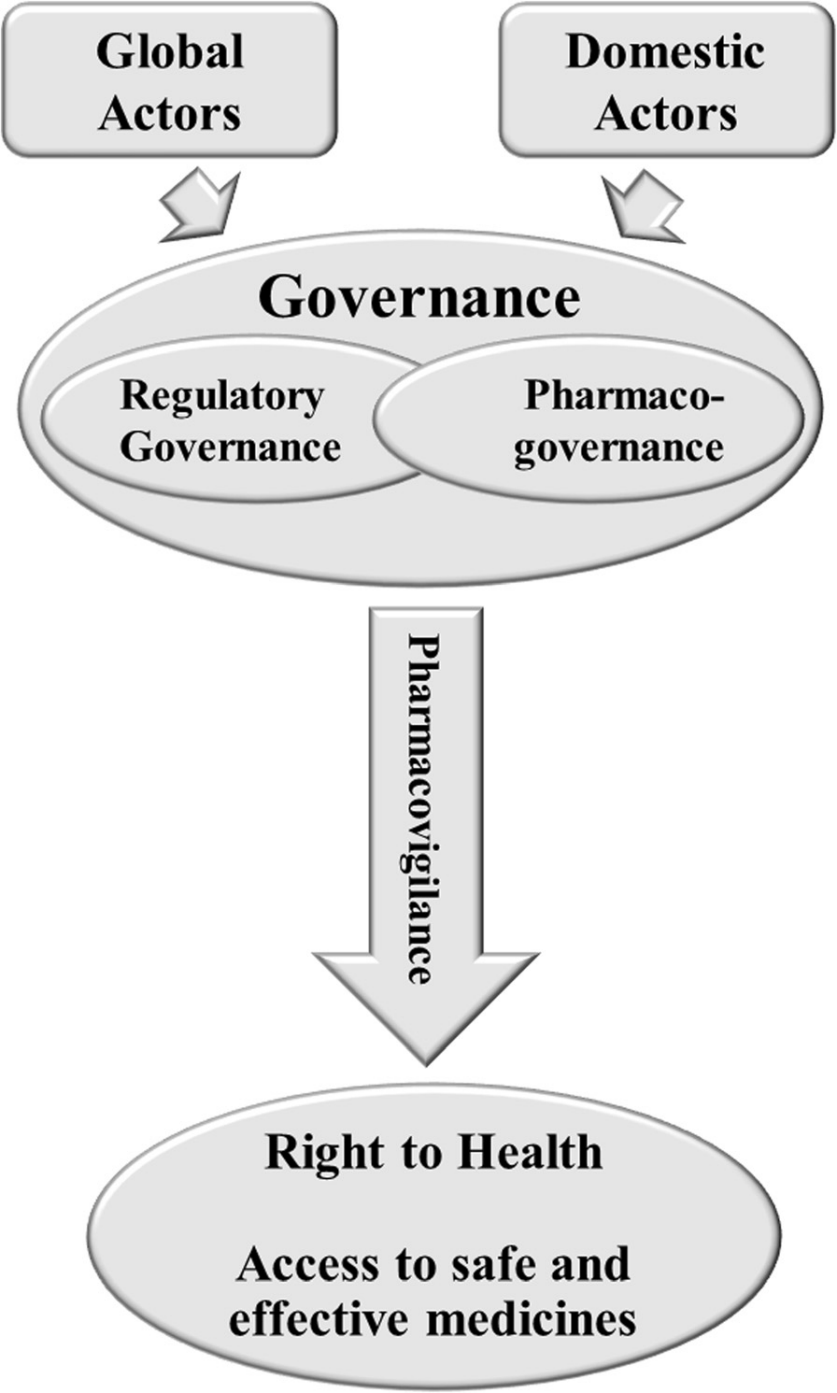
Factors influencing pharmacovigilance in Brazil

The 1990’s was marked by a groundswell of discourse supporting pharmacovigilance by domestic and global actors. Support for pharmacovigilance grew in Brazil’s universities, consumer advocacy groups, drug information centers, and health professional associations during the 1990s [22]. State pharmacovigilance centers and drug information centers (Centros de Informação de Medicamentos) were established in São Paulo, Ceará, Paraná and Mato Grosso do Sul during the period between 1989 and 1998 [16, 22–25]. Pharmacovigilance was also the focus of the IV Brazilian Congress on the Surveillance of Drugs (1997), Conference of Brazilian Society of Hospital Pharmacy I and II, and the 1^st^ Brazilian Seminar on Pharmacoepidemiology [16]. Global actors’ policy ideas during this period, as later described in this paper, served as a catalyst for discussions regarding nationwide pharmacovigilance systems. The disseminated policy ideas influenced state pharmacovigilance initiatives in Brazil [14] (Fig. 1).

The Agência Nacional de Vigilância Sanitária (ANVISA) was established in 1999. ANVISA ’s mandate is to protect and promote population health and ensure access to safe medicines, health products and services [3, 26]. It is one of Brazil’s largest regulatory agencies; overseeing the implementation of aspects of Brazil’s NMP [27, 28]. ANVISA regulates products, sectors and services related to health and numerous areas not directly relevant to pharmaceuticals or medical devices (e.g., foods, tobacco, agricultural chemicals, airports, and border surveillance) although much of its resources are allocated to non-health sectors [26, 29]. It regulates products and services that are valued at approximately 25 percent of Brazil’s gross domestic product [29].

ANVISA’s governance reflects the reform agenda championed by President Fernando Henrique Cardoso beginning in 1995 and continuing throughout his presidency. Cardoso’s endorsement of regulatory oversight led to a surge in the creation of newly structured regulatory authorities [30, 31]. ANVISA’s governance also reflected global actors’ ideas for regulatory governance that were circulated during the 1990s [29, 30].

### The post 2000 period

Brazil’s National Pharmacovigilance System (NPS) was adopted in 2001. The NPS is managed by the Pharmacovigilance Unit [27] and coordinated by ANVISA [27, 28]. The National Center for Monitoring of Medicines (CNMM) was also created in 2001 following the meglumine tragedy (2000) that resulted in hundreds of fatal ADRs [14, 27, 32]. The emergence of yet another incident of serious and fatal ADRs resulting from the use of medicines (e.g. thalidomide in1960) reinforced to the Federal government the need for governing structures and institutional authority over drug safety in Brazil. Today, the CNMM is headquartered in the Pharmacovigilance Unit and is responsible for planning, coordinating and supervising the formulation and implementation of operational guidelines and technical norms for medicines safety, rational use and surveillance.

Like many areas of the health system governance, responsibilities are shared at different levels. Both ANVISA and Brazilian state governments have responsibility for pharmacovigilance. State Centros de Vigilância Sanitária (CVS) are responsible for implementing policy and practices to reduce ADRs and poor quality medicines. Pharmacosurveillance, monitoring drug adverse-effects for signals of safety issues, is carried out by regional pharmacovigilance centers in 193 sentinel hospitals and sentinel pharmacies as part of the Notifying Pharmacies project [33, 34]. The Notifying Pharmacy project (Farmácias Notificadoras), a partnership between the CVS and State Boards of Pharmacy, requires a pharmacist be present during pharmacy operating hours and submit reports of drug-related problems to the CNMM [35].

## Methods

A scoping review of peer reviewed and grey literature from pharmacy, health, political science, and the social sciences, pertaining to global actors (e.g. WHO, Global Fund) and pharmacovigilance, regulatory governance, accountability and transparency in Brazil was conducted for this study of governance and pharmacovigilance in Brazil. The scoping review was used to map the existing literature and gather a holistic picture of pharmacogovernance in Brazil. Cochrane Collaboration guidelines for qualitative research were followed for searching, inclusion, and data extraction [36, 37]. The full search strategy is presented in Table 1 and follows the STARLITE reporting criteria [36]. The acronym STARLITE represents **s**ampling strategy, **t**ype of study, **a**pproaches, **r**ange of years, **l**imits, **i**nclusion and exclusions, **t**erms used, and **e**lectronic sources. Although we narrowly defined the research question, pre-determined inclusion and exclusion criteria, and followed a strategy for data extraction- consistent with a systematic review, we did not apply quality filters and nor formally assess the quality of the literature included in our study- consistent with a scoping review [38].

**Table 1:** Scoping Review structured According to STARLITE Principles

	STARLITE principles
S	Selective **sampling strategy**: Articles selected from pharmacy, health, political science, and the social sciences databases
T	All **types of studies** were included (policy papers, qualitative studies, dissertations)
A	**Approaches:** Subject searching, citation searching, hand-searching, internet searching
R	**Range** (No restrictions): to the beginning of each database—to November 18, 2013
L	No **Limits**
I	**Inclusion**: Global actors and pharmacovigilance, regulatory governance, accountability and transparency in Brazil; **Exclusion**: Studies describing 1) vaccines, herbals or animal studies; 2) pre-market studies (phase I, II, and III); 3) pharmaceutics methods; 4) randomized controlled trials or observational studies pertaining to therapeutics or characterizing drug-specific ADRs or 5) did not describe pharmacovigilance in Brazil
T	**Terms** (See Table 1)
E	**Electronic sources**: Ovid MEDLINE(R), Ovid OLDMEDLINE(R), Ovid MEDLINE(R) In-Process & Other Non-Indexed Citations, Ovid Healthstar, Embase Classic + Embase, International Pharmaceutical Abstracts, International Political Science Abstract, Journals@Ovid Full Text, Embase, LILACS, PubMed, EBSCO, SciELO, GOOGLE Scholar

### Search methods

Search methods included entering search terms into relevant databases, organizational websites (e.g., ANVISA) and hand searching. Sixty-three search terms were entered into 13 relevant databases on November 17-18, 2013 to identify literature pertaining to pharmacovigilance, governance, transparency, specific global actors, the pharmaceutical industry, ANVISA, and civil society (Additional file 1). Acronyms and full text were entered as search terms, such as World Health Organization and WHO. All databases were searched for the same time period which was the beginning date of the database (e.g., OVID [1946] and International Pharmaceutical Abstracts [1970]) through November 18^th^, 2013. Data were only available through October 2013 for some of the databases searched. This date range was selected to capture literature describing global actors’ influence in Brazil in the years prior to the creation of ANVISA up to the date of the search. The databases searched were Ovid MEDLINE(R) 1946 to November Week 1 2013, Ovid OLDMEDLINE(R) 1946 to 1965, Ovid MEDLINE(R) In-Process & Other Non-Indexed Citations November 15, 2013, Ovid Healthstar 1966 to October 2013, Embase Classic + Embase 1947 to 2013 Week 46, International Pharmaceutical Abstracts 1970 to October 2013, International Political Science Abstract 1989 to October 2013, Journals@Ovid Full Text November 18, 2013, Embase 1974 to 2013 November 15, LILACS DATE 1^st^ mentioned to November 18, 2013, PubMed 1^st^ mention to November 18, 2013, EBSCO search Oct 16, 2013 and SciELO 1^st^ mention to Nov 17, 2013. There were no search restrictions. English, Portuguese, and Spanish publications were included.

The **1137** records retrieved were derived from: OVID/Embase (**986**), LILACS (**57**), PubMed (**89**) and EBSCO (**5**). After duplicates were removed **358** records remained. Data from ANVISA, WHO and PAHO websites (**4**) was included.

### Criteria for selecting studies

**Fig. 2:**
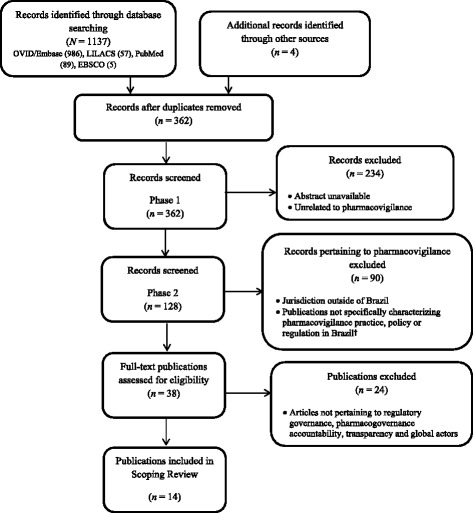
Scoping review flowchart. †Reasons for excluding articles included: descriptive studies of vaccines, herbals/phytopharmaceuticals, nutraceuticals, OTCs, pharmacovigilance interventions, characterization of drug specific ADRs, methods and tools for assessing causality

Two researchers (KM, PC) read through the titles and abstracts (all written in English) to determine relevance to this study. Phase I exclusion criteria comprised publications that described: 1) vaccines, herbals or animal studies; 2) pre-market studies (phase I, II, and III); 3) pharmaceutics methods; 4) randomized controlled trials or observational studies pertaining to therapeutics or characterizing drug-specific ADRs and 5) studies that were retrieved solely because the author was from Brazil, or a Brazilian reference was cited (Fig. 2). In phase II, the full text was read to determine inclusion (Table 2). Full texts written in Portuguese and Spanish were read; then translated into English using Google translate; then re-read before determining inclusion. Publications not meeting inclusion criteria provided background information to contextualize Brazil’s experience with pharmacovigilance.

**Table 2:** Characteristics of literature screened for inclusion or exclusion

Published literature on pharmacovigilance and regulatory governance [*n* = 128]			
Type	Topic	Subject	*n*
A	ADR studies:	A1: Specific drug(s) [43]	65
	Prevalence and Characterization of ADRs	A2: Vaccines, herbals, phytopharmaceuticals, nutraceuticals, Over-the Counter [22]	
B	Theoretical papers (Not Brazil specific)	B1: ADR reporting [3]	14
		B2: Risk communication [1]	
		B3: Regulatory harmonization [1]	
		B4: Global actors’ norms or Global governance [6]	
		B:5 Pharmacovigilance regulatory authority Latin America [3]	
C	Pharmacovigilance practices in Brazil	C1: Industry implementation of pharmacovigilance [2]	35
		C2: Analysis of pharmacovigilance centres, sentinel hospitals & notifying pharmacy ADR reports [9]	
		C3: Pharmacovigilance Systems, regulations, or policies [20]	
		C4: ADR prevention interventions [4]	
D	Regulatory governance, pharmacogovernance and pharmacovigilance	D1: Transparency and/or Accountability [5]	14
		D2: Global actors and Transparency and/or Accountability [3]	
		D3: Regulation, Policy and Law [6]	

### Types of studies included

Fourteen publications met our inclusion criteria (Table 2). The publications that were included characterized: 1) global interventions in Brazil pertaining to governance or pharmacovigilance, 2) ANVISA regulatory governance (e.g., accountability and transparency), and 3) pharmacogovernance in Brazil. All of the publications that met the inclusion criteria were read iteratively by the principal author and data was extracted that was relevant to 1) how global actors, their policy ideas and instruments influenced Brazil’s regulatory governance and pharmacovigilance and 2) how ANVISA’s pharmacogovernance supports pharmacovigilance.

### Data extraction and management

**Fig. 3:**
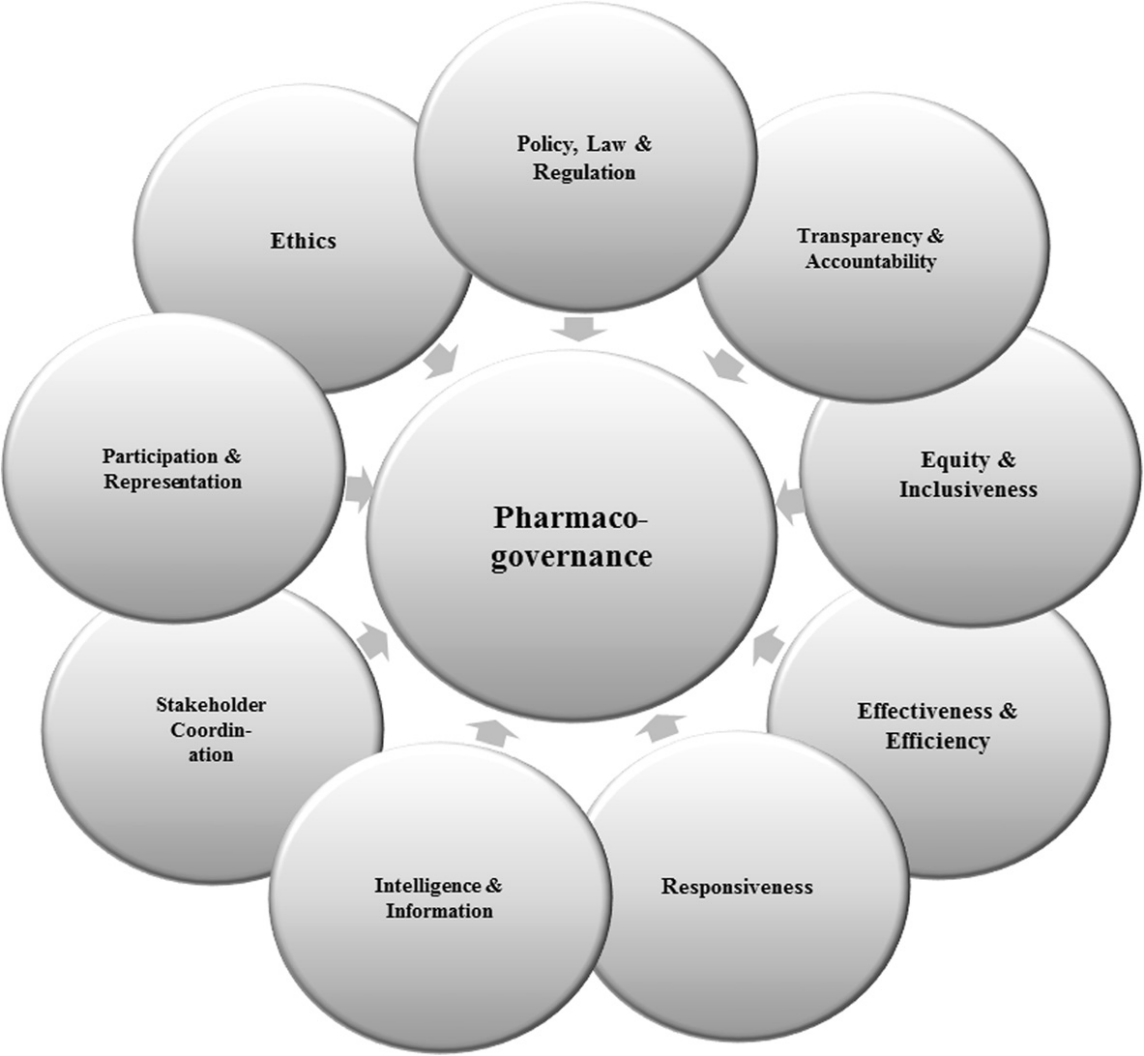
Pharmacogovernance framework

A selective approach to data extraction was employed in this research [37]. Data specifically related to the study question(s) and the pharmacogovernance domains were extracted. A pharmacogovernance framework was used to analyze the relationship between pharmacogovernance and pharmacovigilance. The pharmacogovernance domains were established a priori. Our pharmacogovernance domains were: Policy, Law, and Regulation; Transparency and Accountability; Participation and Representation; Equity and Inclusiveness; Effectiveness and Efficiency; Intelligence and Information; Ethics; Responsiveness; and Stakeholder coordination (Fig. 3). Quality of the literature included in this review was neither prioritized nor formally assessed.

## Results

Our scoping review identified fourteen publications on the topic of governance and ANVISA in Brazil; nine specifically addressed accountability and/or transparency. From this sample, four referenced global institutions. Nearly half of the publications (4) were written by persons internal to ANVISA and described regulation and regulatory reforms in ANVISA. The publications that were analyzed were classified into 4 typologies. The typologies that were found were classified into the following areas: 1) ANVISA’s regulatory reforms and policy instruments; 2) ANVISA’s regulatory governance; 3) global actors’ influence on norms in Brazil pertaining to governance or pharmacovigilance; and 4) ANVISA and the pharmacogovernance domain(s) (Table 3).

**Table 3:** Typology of included literature

Literature typology	Authorship and date	Types of literature	Article summary	Data extracted
Literature describing ANVISA regulatory reforms and policy instruments	ANVISA (2009) ANVISA Stratégias prioritárias da gestão institucional. www.anvisa.gov.br/divulga/noticias/2009/pdf/cartilha_pmg.pdf	Agency report	ANVISA’s strategies and priorities for institutional management	• ANVISA 2008 strategic plan (summary) • Aims of regulatory management reform • The Regulatory Agenda as a policy instrument to strengthening regulatory governance through increased transparency and social participation • ANVISA’s ongoing experience with the regulatory reform
	ANVISA (2009) Gaetani, F., & Albuquerque, K. Capítulo 8: Análise de impacto regulatório e melhoria regulatória	Commissioned report: Regulation and Agency Regulators: Governance and analysis of regulatory impact (Chapter 8)	Regulatory Impact Analysis (RIA) as a policy instrument to improve regulatory management	• Globalization and regulatory reform in Brazil • Rationale for adoption of global actors’ policy ideas • Policy, law and regulations adopted for regulatory improvement • Characterization of RIA as a policy instrument to improve the regulatory decision-making • ANVISA’s Regulatory Agenda as a policy tool for transparency • Social participation as a mechanism for transparency
Literature describing ANVISA’s regulatory governance	ANVISA (2009)	Commissioned report: Regulation and Agency Regulators: Governance and analysis of regulatory impact (Chapter 5)	Factors influencing the development of regulatory authorities in Brazil	• Characterization of the political and economic conditions influencing regulatory reforms • Description of enabling legislation for regulatory reform of 10 agencies- including ANVISA
	ANVISA (2009) de Mello, D. R., & Ramalho. Capítulo 11: Boas práticas regulatórias: previsibilidade e transparência na Agência Nacional de Vigilância Sanitária	Commissioned report: Regulation and Agency Regulators: Governance and analysis of regulatory impact (Chapter 11)	Factors influencing ANVISA’s governance and effectiveness. ANVISA’s best practices for regulatory management to improve accountability and transparency.	• Global actors’ influence on ANVISA’s regulatory governance • ANVISA’s characteristics and scope • Relationship between ANVISA’s governance, accountability and transparency
Literature describing global actors’ influence and norms in Brazil pertaining to governance or pharmacovigilance	ANVISA (2009) Cruz, V. Capítulo 2: Estado e regulação: fundamentos teóricos	Commissioned report: Regulation and Agency Regulators: Governance and analysis of regulatory impact (Chapter 2)	Factors influencing the development of regulatory authorities in Brazil	• Global actors’ influence on regulatory reform and ANVISA’s governance structure • Rationale for adoption of global actors’ policy ideas
	Dainesi, S. (2005).	Journal Article	Implementation of pharmacovigilance in Brazil	• Characterization of pharmacovigilance pertaining to efficiency and transparency • Recommendations for supporting a culture of disclosure to advance pharmacovigilance
	PAHO. (2011)	Commissioned report:	Description of PAHO best practices for pharmacovigilance	• PAHO norms for pharmacovigilance
Literature describing ANVISA and the pharmacogovernance domain(s)	Biehl, J., et al. (2009). Princeton University, Princeton	Commentary	Description of the growing trend of litigation to gain access to medicines in the context of incomplete knowledge of the safety of medicines.	• Need for transparency in drug approval process and placement of drugs on SUS formulary.
	Cruz, V. (2010)	Journal Article	ANVISA is used as a case study of institutionalized mechanisms for accountability and transparency	• Civil society participation as a mechanism to increase regulatory accountability and transparency • Global actors’ influence on ANVISA’s regulatory governance • ANVISA’s management contract as an instrument of accountability
	Freitas, M. & Romano-Lieber, N. (2007)	Journal Article	Laws pertaining to pharmacovigilance and industry	• Laws requiring ADR reporting by industry • Laws requiring industry pharmacovigilance departments • Evaluation of the effectiveness of laws
	Gava, C., et al. (2010)	Journal Article	Medicines registration process for new and generic drugs	• Characterization of Brazil’s drug registration process • Description of processes lacking transparency
	Miranda, A. (2010).	Dissertation	ANVISA’s experience with transparency in regulatory management	• Characterization of the conditions leading to drug safety reforms • Characterization of ANVISA’s scope and mandate • Social participation and transparency in regulatory management • Characterization of the effectiveness of ANVISA’s actions to include social participation in decision making
	Mastroianni PC, Lucchetta RC. (2011)	Journal article	ANVISA and the drug approval process	• ANVISA’s role in protecting medicines safety • Drug registration and re-registration requirements
	Pereira, M. F. (2010)	Thesis	Social participation and community health councils	• Characterization of social participation as a mechanism of governance in Brazil
	Prat, A. G. (2013)	Journal Article	Laws pertaining to drug approval	• ANVISA requirements for drug registration in Brazil
	Silva, G. H. (2011). IV Congresso CONSAD de Gestao Publica.	Conference paper	ANVISA’s experience using the Regulatory Impact Analysis	• Global actors’ influence on ANVISA’s regulatory reforms with emphasis on transparency and accountability • Characterization of ANVISA’s Regulatory Process Improvement Programme including the Regulatory Agenda • Analysis of the implementation of the Regulatory Agenda • Characterization of effectiveness and remaining gaps
	Vashisth, S., Singh, G., & Nanda, A. (2012)	Journal Article	Comparative study of pharmacovigilance in Brazil, Russia, India and China	• Requirements for drug approval • Characterization of effectiveness and gaps in pharmacovigilance • Recommendations to strengthen pharmacovigilance (e.g., enforcement)

### Literature describing ANVISA’s regulatory reforms and policy instruments

ANVISA was one of the earliest regulatory authorities to take up reforms aimed to strengthen regulatory management processes [21]. The ANVISA Regulatory Process Improvement Programme or Good Regulatory Practices Program (PRO-REG) required mandatory implementation of a Regulatory Agenda [10].

ANVISA’s priority areas are adopted into an agenda that is established through participatory governance whereby the public is invited to participate in a consultation process [10]. The general population and industry may participate in the public consultation. The Regulatory Impact Analysis (RIA) is another policy instrument to improve and strengthen the regulatory system [10, 39]. The RIA is described as a tool for accountability and transparency because it evaluates policy effectiveness, efficiency and responsiveness in meeting ANVISA’s regulatory agenda [10, 21]. According to Ramalho (2009 p8), PRO-REG and RIA are tools for ANVISA to create an *‘institutional environment favourable to social and economic development of the country.*

### Literature describing ANVISA’s regulatory governance

ANVISA was established as an independent regulatory body with administrative and fiscal autonomy, under contract with the Ministry of Health (MoH) [26, 29]. It also receives funding annually from pharmaceutical company registration and drug registration fees.

ANVISA is governed by a 5-member Collegiate Board of Directors that is accountable for the agency’s activities [26]. Its Advisory Board includes representation from industry, the scientific community, government and the public.

Brazil’s Federal Constitution gave legitimacy to public stakeholder engagement in decision-making spaces. Participatory governance was endorsed as a *‘strategy for strengthening governance and the legitimacy of regulatory action in the country’* ([10], p1) yet, consumer interests are represented by only two of ANVISA’s 12-member board (Instituto Brasileiro de Defesa do Consumidor and Fundação Procon/São Paulo) [8]. ANVISA’s Ombudsmen, appointed by the Minister of Health and approved by president of Brazil, was established to respond to citizen issues; providing another mechanism to the public interests to be voiced.

### Literature describing global actors’ influence on norms in Brazil pertaining to governance or pharmacovigilance

Brazil’s pharmacogovernance, especially the adoption of regulatory agencies, drug safety policy and pharmacovigilance norms, has been shaped by national and transnational actors (Additional file 2) [2, 8, 14, 21, 29, 30, 40, 41]. They endorsed the establishment of National Regulatory Authorities (NRAs) e.g., ANVISA. Global actors, such as the Pan American Network for Drug Regulatory Harmonization (PANDRH) Working Group on Pharmacovigilance, endorsed *‘…qualification of the NRAs in the Region in accordance with criteria established by PAHO/WHO in order to establish reference Regulatory Authorities…’,* in Brazil and throughout Latin America to achieve access to quality, safe, and efficacious medicines ([42], p1). Brazil was recognized by PAHO as one of 5 regional reference authorities [43].

Domestically, Carlos Luis Bresser-Pereira, Brazil’s Minister of Federal Administration and Reform of the State (1995-1998) championed regulatory reform to address *‘bureaucratic administration [that] is slow, [and has] little or nothing geared to meet the demands of citizens’* [31, 44]. Silva (2011) and Ramalho (2009 p127) posited that regulatory reform in Brazil was also motivated by the desire to harmonize regulatory practices with global norms ‘*especially as regards [to] conformation of bureaucracy and its interaction with the "outside world"*’ and to increase Brazil’s acceptance into ‘*the circle of countries with a modern regulatory system*’([30], p56).

According to Silva (2011 p1) Brazil’s National Regulatory Agenda and ANVISA reforms have *‘mirrored most developed countries’*. ANVISA’s Good Regulatory Practices Program (PRO-REG) is modeled after FDA, Health Canada, Australia Therapeutic Goods Administration, UK Medicines and Healthcare Products Regulatory Agency, and Portugal’s Instituto Nacional da Farmácia e do Medicamento [10]. The PRO-REG has incorporated OECD norms for regulatory impact analysis and WHO principles for Good Governance in Medicines (GGM) [45]. Although Brazil is not an active participant in the GGM programme, ANVISA has adopted many WHO/PAHO norms pertaining to pharmacovigilance. Specific norms include: good governance for supply chain management, code of ethics to prevent corruption, good manufacturing processes spontaneous ADR reporting and sentinel reporting sites [11, 15, 46, 47].

### Literature describing ANVISA and Pharmacogovernance

We analyzed ANVISA’s pharmacogovernance in nine core domains: Policy, Law and Regulation; Transparency and Accountability; Responsiveness; Participation and Representation; Equity and Inclusiveness; Effectiveness and Efficiency; Intelligence and Information; Ethics; and Stakeholder coordination.

#### Policy, Law and Regulation

Brazil has well defined ‘policy, law, and regulation’ to enable pharmacovigilance. Pharmacovigilance is integrated into Brazil’s national health care system. Existing laws grant the agency authority to regulate drug registration, ADR reporting, approve patent applications and drug pricing. Pharmaceutical companies desiring product registration in Brazil must submit proof of safety and efficacy [3]; an EMA Certificate of Medicinal Product or Certificate of Pharmaceutical Product issued by FDA or country of origin; and may undergo manufacturing site inspection to assure ANVISA’s Good Manufacturing Practices are observed. Product registration must be renewed every five years [3]. The renewal process requires submission of ADR reports, complaints, technical reports of therapeutic ineffectiveness, pharmacovigilance data, and product long-term stability studies [3]. Generic drug registration requires submission of tests for pharmaceutical equivalence and bioavailability to their reference drug [3].

Drug manufacturers are required to establish a corporate pharmacovigilance program. Freitas and Romano-Leiber (2007) found that despite resolution RDC No. 4, Article 3 (2009) fewer than half the companies responding to their survey (20) had implemented a program. Thirteen companies that implemented a pharmacovigilance program were multinational corporations and 7 were domestic companies [48]. Market authorization holders are required by Federal law (n° 6,360/76 article 79) to report ADRs associated with their drugs to the competent health authority [33, 49], however Freitas and Romano-Lieber (2007) found that few domestic companies provided regular training for reporting ADRs. Compliance with international regulatory requirements and international harmonization was the rationale given for reporting by 25 % of the pharmaceutical companies surveyed [48].

Despite policies, laws, and regulations intended to support ANVISA’s mandate to ensure access to safe medicines, health products and services (Additional file 2), our study found literature describing a lack of standardization and regulation of medicines prior to the adoption of Brazil’s NMP [50] that still persists today.

#### Transparency and accountability

‘Transparency’ in the public pharmaceutical sector is defined as openness in sharing information. It is a *‘principle whereby those affected by administrative decisions should be informed, and it is the duty of civil servants, managers and trustees to act visibly, predictably and understandably*’ ([6], p162). Information is publicly and easily accessible when regulatory decision making is transparent. Transparency aids in building understanding and trust from healthcare professionals in regulatory decisions and risk minimization measures [51]. We define accountability as taking responsibility for postmarket drug safety policy outcomes.

Accountability and transparency were described as agency values on ANVISA’s website [26]. Norms for transparency were codified by Ministerial Decree n°5.482/05. ANVISA’s regulatory agenda and management contract were described as instruments of accountability (Cruz 2010 and Silva 2011). Both aimed to address past issues that included a ‘*lack of systematization and standards for the regulatory process;… lack of predictability of regulatory actions; …and inadequate mechanisms for transparency… and participation’* ([10], p3). ANVISA’s 3-year management contract was described as a mechanism of administrative review of the agency’s performance [41]. The requirement for ANVISA to submit reports to its advisory board, the MoH, National Health Council and competent authorities to account for its activities was described as another mechanism of accountability. The integration of public participation in consultations and hearings to debate ANVISA’s regulatory agenda was also described in the literature as a mechanism for transparency and accountability [8, 52].

The literature describing transparency, accountability in ANVISA’s administrative procedures has been contested. We identified literature that emphasized how transparency was needed in the drug approval process, drug surveillance and regulatory control of medicines [2, 9]. ANVISA’s lack of transparency was noted in the 13th National Conference on Health report [8]. Gava et al. (2010) argued transparency was lacking in ANVISA’s approval of ‘me-too’ drugs that have little benefit over drugs currently marketed. A ‘me-too’ drug is a new molecular entity or biological equivalent, structurally similar to an existing drug (e.g., anti-cholesterol drugs atorvastatin and pravastatin). Gava et al. (2010) have recommended a more transparent registration process whereby data is publicly available to inform consumers, health professionals and health managers, about the true benefits and risks of drug treatment.

A behavioural impediment to a culture of transparency and disclosure was also described in the literature. Dainesi (2005) found that health care professionals’ reluctance to report errors, adverse events and treatment failures was an impediment to pharmacovigilance. ANVISA, industry and academia must each promote *‘values that should guide corporate governance’*: a culture of transparency, justice, overall compliance with regulations, and accountability ([53], p186).

#### Participation and representation

The pharmacogovernance domain ‘participation and representation’ pertains to public representation and involvement in decision making at regulatory authority and government public meetings to establish the regulatory agenda and rules for postmarket drug safety. ANVISA’s Regulatory Agenda is determined annually through what is reported to be a participatory process. The Brazilian constitution and laws support social participation to get public input in regulatory decision making.

We found literature that suggested that the general public was under represented in public decision making spaces (e.g., public forums). Public representation in Municipal Councils was described as largely comprised of citizens with higher education and income [8]; with lesser representation of minority and marginalized groups [54]. Miranda (2010) found a gap in public knowledge of spaces for citizen participation. Whereas ANVISA described the Ombudsman's Office and the telephone exchange as spaces for public input some key informants did not know how to use the services (e.g. where to submit a report). Additionally, Miranda (2010) found that key informants incorrectly identified the National System of Controlled Products Management (SNGPC) and Sistema de Notificações para a Vigilância Sanitária (NOTIVISA) as spaces for public participation. NOTIVISA is Brazil’s online system for reporting ADRs. Consumers may not submit reports to this online system. Only industry, health professionals, hospitals and pharmacies are permitted to submit reports to NOTIVISA.

#### Equity and inclusiveness

We define ‘inclusiveness’ as spaces for public participation in pharmacovigilance policy setting that are accessible to all segments of the population*.* ANVISA working papers describe agency actions to increase spaces for social participation in ANVISA’s decision making [8, 10, 39, 41, 52]. Citizen consultation in public hearings is required by ordinance prior to the adoption of new regulatory standards or rules changes [8, 52]. The literature suggests that although public policy and regulation in Brazil aims to encourage inclusive decision making, equity and inclusiveness has not yet been realized [8, 54].

Miranda (2010) and Pereira (2010) found gaps in the public’s capacity to participate in ANVISA’s decision making spaces that impede inclusive governance. Although advance notice of public meetings is posted to ANVISA’s website disparities exist in internet access. Up to 65 % of the population in some Brazilian states has limited to no internet access [8, 55]. Online notification fails to reach audiences without computer access. Meetings scheduled at times and locations that are not readily accessible to the public limits inclusion. The literature suggests that inclusion in ANVISA’s public consultation for its Regulatory Agenda is asymmetric with greater participation by industry and wealthy individuals. *‘Information asymmetry between the government regulated sector and society’* compromises transparency and equity ([8], p78-9).

We define ‘equity’ as economic and social resource allocation to ensure that all regions within the country have access to safe medicines and resources to detect and act on drug safety signals. Pharmacovigilance coverage in all regions is a measure of equity. The literature suggests that resources to monitor and assess drug safety are not distributed equitably nationwide. Although sentinel hospitals are located throughout the country, the number of sentinel sites is greatest in the most highly resourced and densely populated southeast region (includes São Paulo, Rio de Janeiro, and Minas Gerais) and the least in rural, less populated north and central-west regions with poverty levels up to 42 percent [55]. While ANVISA’s Notifying Pharmacies project has the potential to expand pharmacosurveillance, participation has been low [33, 56].

#### Effectiveness and efficiency

Pharmacovigilance policy, law and regulations are defined as effective when they benefit patient safety. Actions to improve pharmacovigilance are efficient when they are undertaken in a timely manner. We found gaps in the literature pertaining ANVISA’s analysis of the effectiveness of pharmacovigilance policies and their capacity to monitor compliance with policy, law and regulation pertaining to postmarket drug safety.

Ramalho (2009) suggested that ANVISA’s effectiveness has been challenged by: 1) fragmented establishment of norms; 2) a culture of disregard for rules of the State; 3) unnecessary or overlapping regulations; 4) ineffective monitoring and enforcement; and 5) poor design and/or implementation of norms leading to high costs for compliance. ANVISA’s diverse portfolio was described as too expansive to “*effectively monitor a pharmaceutical market with the size and growing demand of Brazil”* ([33], p141). Vashisth et al., (2012) argued that stronger enforcement mechanisms were needed to strengthen pharmacovigilance, particularly in regards to generic drugs registered before 2003 (prior to proof of bioequivalence requirements), although the extent of the problem of substandard generics was not reported.

#### Intelligence and information

The pharmacogovernance domain ‘intelligence and information’ pertains to mechanisms that exist to improve communication among the national regulatory authority, state pharmacovigilance centers, healthcare professionals, policymakers, patients and the general public with respect to medicine safety. Risk communication about drugs with real and potential safety issues is important for enabling the safe use of medicines [51].

Our analysis of the literature describing ANVISA’s pharmacogovernance in the domain intelligence and information is limited. We found only three abstracts that described risk communication in Brazil. Two abstracts described ADR reporting mechanisms- ANVISA’s website, sentinel hospitals, and the CNMM [57, 58]. The third described the importance of training health professionals to report ADRs [59]. None of the abstracts described the process by which ANVISA communicates information about safety signals to the states or municipalities. Given the inequities in the sentinel reporting site distribution, it is anticipated that corresponding inequities exist in risk communication.

Incompatibility between databases in Brazil was described as an impediment to data sharing of ADRs reported for medicines and immunizations [personal communication 2014]. Brazil does not submit case safety reports to the Uppsala Monitoring Centre using the UMC global reporting format. Moreover, Brazil’s domestic ADR reporting form collects less information than 13 countries studied [60].

#### Ethics

‘Ethics’ is defined as respect for justice, autonomy, non-maleficence, and beneficence to safeguard patient interests, right to safe medicines and health. Miranda (2010) argues that ANVISA has a dual mandate: to increase the competitiveness of domestic industries under its purview and protect population health. The dual mandate undermines beneficence and patients’ rights to safe medicines and health. The literature challenges the assumption that ANVISA can balance incompatible societal interests in patient safety and industry interests [8, 10]. Gava et al. (2010 p3410), suggest that while *‘…health authorities should act as mediators between the interests of drug manufacturers and the needs of public health… [they have a] duty to protect health’*. Access to safe medicines may be compromised by ANVISA’s policy to evaluate the potential impact of regulatory action with regard to national competitiveness [10]. Miranda (2010) and Silva (2011) concur that ANVISA must reconcile its dual mandate to *‘better withstand the volatile nature of conflicts of interest in relations of production and consumption, seeking to strengthen its regulatory role…’* ([10], p20).

#### Responsiveness

The pharmacogovernance domain ‘responsiveness’ is defined as promptly acting to address drug safety issues and enact pharmacovigilance policies/regulations. We found a gap in the literature pertaining to ANVISA’s responsiveness in addressing drug safety issues. Although we are aware that the agency has posted drug safety alerts on its website, we were unable to find literature of Regulatory Impact Analyses conducted of ANVISA’s risk communication policies for responding to drug safety issues. The literature regarding RIA was primarily descriptive; assessing whether ANVISA acted on an agenda item [10] rather than the impact of specific policies or regulation.

The first agenda items directly relevant to pharmacovigilance were added to the 2013-2014 agenda, five years after the Regulatory Agenda policy was implemented [61]. They pertain to requirements for companies to communicate registration changes, product labeling risk communication, and rational use of medicines.

#### Stakeholder coordination

The pharmacogovernance domain ‘stakeholder coordination’ describes actions by ANVISA and global actors to coordinate activities aimed to strengthen pharmacovigilance. The literature was searched for evidence of stakeholders’ efforts to coordinate initiatives and/or resources to strengthen postmarket drug safety. We found a gap in the literature regarding stakeholder coordination for the purpose of enabling pharmacovigilance. We identified literature describing global and domestic actors’ interventions, policy preferences and norms in Brazil, but not examples of stakeholder coordination pertaining to any specific pharmacovigilance intervention.

## Discussion

The present study contributes to the literature on pharmacogovernance and the relationship between governance, pharmacovigilance and global institutions in Brazil. The few articles published are written in Portuguese limiting the transfer of knowledge about Brazil’s experience with pharmacogovernance. This is the first English language review of which we are aware.

Understanding the relationship between ANVISA’s governance and pharmacovigilance is important. Pharmacogovernance embraces a culture of postmarket drug safety. It assures that governing structures, policy instruments, authority to implement and enforce norms, policies and processes preserve societal interests for patient safety and protection from adverse drug events.

Regulatory governance and pharmacogovernance that best supports pharmacovigilance is still being debated globally [41, 62–65]. Questions regarding governing structures, authority to implement and enforce norms, policies and processes to mitigate ADRs, the regulator-industry relationship, scope of regulatory authority, reliance on industry-produced studies, mechanisms for independent review and accountability for decision-making remain.

Our study found that the literature written by those internal to ANVISA was mostly favourable to the agency’s efforts to advance a culture of transparency and accountability. One study, written by an author external to ANVISA, suggested that greater transparency was needed regarding drug registration, reauthorization and ADR reporting to benefit patients served by Brazil’s National Health System (Sistema Único de Saúde [SUS]).

Transparency confers legitimacy, increases accountability in decision-making and is a basic requirement of good governance [8, 41]. The absence of transparency obfuscates the ability to identify whose interests are served by policy preferences adopted.

We found Brazil’s pharmacogovernance was strongest in the domain of policy and law. Existing regulations should effectively enable access to safe medicines [43]. Gaps in other domains however disenabled postmarket drug safety and have led to regional disparities in pharmacovigilance between highly resourced states and under resourced Brazilian states.

Our findings regarding the pharmacogovernance domain ‘intelligence and information’ suggest that signal generation and risk communication may be impeded in Brazil. ‘Ethics’ is challenged by ANVISA’s excessively broad purview over disparate sectors (e.g. pharmaceuticals to airports) and dual industry-health mandate that threaten strong pharmacovigilance policies. Industrial interests and public health interests are not typically aligned. Conflicts arising from industry accountability to shareholders have been shown to create tensions that impede pharmacovigilance [4, 66, 67]. With ANVISA’s dual mandate, the balance between medicines safety, accessibility, and economic development, is largely unachievable. This could be mitigated by addressing concerns about transparency in drug approval and re-approval decisions raised in the literature.

ANVISA’s Regulatory Agenda and Regulatory Impact Analysis could advance pharmacogovernance. To be sure, since this study was conducted new norms and revisions of existing norms for medicines have been added to the agenda. However, ANVISA has not undertaken analysis of the impact of its pharmacovigilance system nor analyzed the effectiveness of parallel reporting systems between states and the federal government. Regulatory impact analyses of ANVISA’s stakeholder participation have not been conducted either. Greater public representation and participation would improve accountability and could in principal hold regulators and drug companies to account for their decisions pertaining to pharmacovigilance. Spaces for social participation must be accessible to be effective and our research found that strategies were needed to make public representation more inclusive [8, 54].

Public participation in setting ANVISA’s Regulatory Agenda could enable the adoption of policies that focus on patients’ drug safety and advance pharmacovigilance. The absence of norms for inclusive stakeholder participation in decisions regarding pharmacovigilance is troubling and ANVISA’s expansive definition of ‘public’ opens public forums to industry as well as consumers; diluting the voice of consumers. This asymmetry may disenable decisions that improve equity in pharmacovigilance and compromise transparency [8, 41].

ANVISA’s current governance and pharmacovigilance norms reflect global and domestic actors’ policy ideas. This has benefitted pharmacogovernance with respect to norms for pharmacovigilance systems, infrastructure, drug surveillance and regulatory accountability. Global actors’ influence on pharmaceutical policy has not always aimed to advance drug safety and access to medicines; therefore, Brazil has exercised autonomy in determining which norms to adopt. An example is Brazil’s decision to retain its 5-year product renewal requirement, unlike the EMA, which reversed its drug reauthorization policy to adopt the US model of continuous reauthorization [3, 68]. Reauthorization permits periodic reassessment of drug registration that informs decisions to require post authorization studies, market withdrawal, or conditional re-approval. It shifts the onus to drug companies to show cause for why drug registration should be reauthorized rather than regulatory justification for why registration changes may be needed.

Policy uptake is strongly conditioned by country specific contexts including national traditions including the pattern of government-industry relations [21, 63]. Brazil’s prior confrontation with the World Trade Organization over the TRIPS Agreement, may explain its reluctance to adopt all global actors’ policy ideas. More accountability for decision-making is desirable to assure public interests are protected from influence by unelected regulatory authorities (e.g. World Trade Organization, International Conference on Harmonisation of Technical Requirements for Registration of Pharmaceuticals for Human Use).

## Conclusion

Our findings suggest that pharmacogovernance that addresses 1) regional disparities in the use of policy instruments and distribution of resources for monitoring and assessing drug safety nationwide, 2) federal and state incoordination of pharmacovigilance regulations, 3) asymmetric representation in public consultation for ANVISA’s regulatory agenda and which 4) disaggregates ANVISA’s health and commercial sector regulatory oversight is needed. Disaggregating ANVISA’s regulatory oversight of health and commercial sectors would mitigate conflicts of interests and disincentives to adoption, implementation and enforcement of strong pharmacovigilance policies.

Further research is needed to better understand the relationship between pharmacogovernance and postmarket drug safety in decentralized, federal systems especially pertaining to accountability and equity. Scope for further research includes studies to answer: 1) How do decentralized state bodies, responsible for the implementation of pharmacovigilance policies, interact with the central regulatory authority policymakers?, 2) Who is accountable for pharmacovigilance where decentralized governance exists? and 3) How is accountability to consumers, industry, the Ministry of Health and global stakeholders balanced?

The literature on pharmacogovernance establishes that, in Brazil, investments in pharmacogovernance processes have generated significant improvements in patient health through a number of mechanisms, including transparency, accountability, policy, law and regulation. The literature also points to a number of mechanisms by which pharmacogovernance may improve health that have not developed as fully in Brazil compared with other nations. Three such mechanisms include, first, comprehensive ethics processes for review of clinical trials for new drugs, proposals for post authorization safety studies and the distribution of drugs; second, the use of new data and analytics to reveal the prevalence of disease and to administer medical resources where they are urgently needed; and third, to improve the engagement of diverse stakeholders in decision-making about resource allocation for pharmacovigilance.

## Additional files

Additional file 1:
**Search terminology.** (DOCX 52 kb) Additional file 2:
**Global institutions pharmacovigilance norms adopted in Brazil.** (DOCX 72 kb)
