# Supplementary material for: Governance and pharmacovigilance in Brazil: a scoping review
Source: J Pharm Policy Pract. 2016 Feb 8;9:3. doi: 10.1186/s40545-016-0053-y (PMC4746882; doi:10.1186/s40545-016-0053-y)
Supplement: Additional file 1: — Search terminology. (DOCX 52 kb) [file 40545_2016_53_MOESM1_ESM.docx]

**Appendix 1. Search terminology**

| 1. Non-state actor and priorities and pharmacovigilance and Brazil 2. Non-state actor and pharmacovigilance and governance and Brazil 3. Non-state actor and transparency and pharmacovigilance and Brazil 4. Transnational actor and priorities and pharmacovigilance and Brazil 5. Transnational actors and pharmacovigilance and governance in Brazil 6. Transnational actor and priorities and transparency and pharmacovigilance and Brazil 7. Intergovernmental priorities and pharmacovigilance and Brazil 8. Intergovernmental agency and pharmacovigilance and governance and Brazil 9. Intergovernmental agency and transparency and pharmacovigilance and Brazil 10. Global governance and pharmacovigilance in Brazil 11. Global governance and pharmacovigilance and governance and Brazil 12. Global governance and transparency and pharmacovigilance and Brazil 13. International institutional influence and pharmacovigilance and Brazil 14. International institutional influence and pharmacovigilance governance and Brazil 15. International institutional influence and transparency and pharmacovigilance and Brazil 16. Pharmacovigilance and Brazil 17. Pharmacovigilance and Brazil and transparency 18. Pharmacovigilance and Brazil and governance 19. Pharmacovigilance and Brazil and transparency and governance 20. Pharmacovigilance and Brazil and corruption 21. Pharmacovigilance and Brazil and Global Fund 22. Pharmacovigilance and Brazil and WHO 23. Pharmacovigilance and Brazil and World Health Organization 24. Pharmacovigilance and Brazil and Gates Foundation 25. Pharmacovigilance and Brazil and DfID 26. Pharmacovigilance and Brazil and international development | 1. Pharmacovigilance and Brazil and World Bank 2. Pharmacovigilance and ANVISA 3. ANVISA and Global Fund 4. ANVISA and World Health Organization 5. ANVISA and WHO 6. ANVISA and World Health Organization and pharmacovigilance 7. ANVISA and Gates Foundation 8. ANVISA and ICH 9. Pharmacovigilance and Brazil and ICH 10. Pharmacovigilance and Brazil and Fiocruz 11. Pharmacovigilance and Brazil and DNDi 12. Pharmacovigilance and Brazil and USAID 13. Pharmacovigilance and Brazil and PAHO 14. Pharmacovigilance and Brazil and Sanofi 15. Pharmacovigilance and Brazil and Merck 16. Pharmacovigilance and Brazil and PEPFAR 17. Pharmacovigilance and Brazil and UK Department for International Development 18. ANVISA and corruption 19. ANVISA and transparency 20. ANVISA and governance 21. Medicine safety and Brazil and policy 22. Drug safety and Brazil and policy 23. Drug safety and Brazil and governance 24. Drug safety and Brazil and transparency 25. Drug safety and Brazil and “pharmaceutical industry” 26. Drug safety and Brazil and pharma 27. Pharmacovigilance and Brazil and policy 28. Pharmacovigilance and ANVISA and policy 29. Pharmacovigilance and Brazil and policy and World Health Organization 30. Pharmacovigilance and Brazil and policy and Gates Foundation 31. Pharmacovigilance and Brazil and policy and World Bank 32. Pharmacovigilance and Brazil and policy and Fiocruz 33. Pharmacovigilance and Brazil and policy and Global Fund 34. Drug safety and Brazil and policy and Global Fund 35. Drug safety and Brazil and civil society 36. Pharmacovigilance and Brazil and PAHO 37. Pharmacovigilance and Brazil and Pan American Health Organization |
| --- | --- |
