# Supplementary material for: Governance and pharmacovigilance in Brazil: a scoping review
Source: J Pharm Policy Pract. 2016 Feb 8;9:3. doi: 10.1186/s40545-016-0053-y (PMC4746882; doi:10.1186/s40545-016-0053-y)
Supplement: Additional file 2: — Global institutions pharmacovigilance norms adopted in Brazil. (DOCX 72 kb) [file 40545_2016_53_MOESM2_ESM.docx]

**Appendix 2. Global institutions pharmacovigilance norms adopted in Brazil**

| **Norm** | **BRAZIL** | **WHO** | **UNAIDS** | **PAHO/ PANDRH** | **Global Fund** | **OECD** |
| --- | --- | --- | --- | --- | --- | --- |
| Risk identification and assessment |  |  |  |  |  |  |
| UMC member | 2001  (62^nd^ member) | X^b^ | X^b^ | X^c^ |  |  |

| Spontaneous reporting^[[1]](#endnote-1)^ | X | X^a^ | X^b^ | X^c^ | X |  |
| --- | --- | --- | --- | --- | --- | --- |
| Cohort Event Monitoring^[[2]](#endnote-2)^ | --- | X^a^ | X^b^ |  |  |  |
| Targeted Spontaneous Reporting^[[3]](#endnote-3)^ | --- | X^a^ | X^b^ |  |  |  |
| Signal generation & data management |  |  |  |  |  |  |
| Database to receive, collate, manage, and analyze ADR reports | X | X^b^ | X^b^ | X^c^ |  |  |
| ADR reporting forms | X | X^b^ | X^b^ |  |  |  |
| Pharmacovigilance system & infrastructure |  |  |  |  |  |  |
| National Regulatory Authority  (ANVISA) | Law 9,782/99  (1999) | X^b^ | X^b^ | X^c^ | X^a^ |  |
| National pharmacovigilance system | 1999 | X^b^ | X^b^ | X^c^ |  |  |
| Brazilian National Medicines Monitoring Centre (CNMM) | Ministerial decree No. 696, of 5/7/2001  (2001) | X^b^ | X^b^ | X^c^ |  |  |
| Center for Drug Surveillance (NVF)- Health Surveillance Center (CVS) of the São Paulo Dept. of Health | 1989 |  |  |  |  |  |
| Pharmacovigilance Unit (UFARM)  (Coordinates the NPS and houses the National Drug Monitoring Centre) | 1999-2001 | X^b^ | X^b^ | X^c^ |  |  |
| Sentinel reporting sites | Hospital/ pharmacy | X^b^ | X^b^ |  |  |  |
| Postmarket surveillance/ ADR reporting guidelines | --- | X^b^ | X^b^ | X^c^ | X |  |
| Adverse events management guidelines | --- | X^b^ | X^b^ |  | X |  |
| Pharmacovigilance communication |  |  |  |  |  |  |
| Risk communication guidelines | RDC n^o^55, article 1 | X^b^ | X^b^ | X^c^ |  |  |
| Rational use of medicines | --- | X^b^ |  | X^c^ |  |  |
| Human resources |  |  |  |  |  |  |
| Pharmacovigilance integrated into national curricula | --- | X^b^ |  |  | X |  |
| Pharmacovigilance expertise | X | X^b^ | X^b^ | X^c^ | X |  |
| Access to training in the country and abroad | X | X^b^ | X^b^ |  | X |  |
| Policy, law, and regulations |  |  |  |  |  |  |
| National Medicines Policy | Ordinance 3.916  1998 |  |  |  |  |  |
| National drug surveillance | Ministerial Decree  No. 696/01 2001 |  |  |  |  |  |
| Federal Constitution  The Organic Law of Health and National pharmacovigilance policy | 1988  Law 8,080/90  1991 |  |  |  |  |  |
| Mandatory postmarket surveillance of pharmaceuticals and  industry reporting | Law 6.360 article 79  1976  RDC n^o^55, article 1 |  |  | X^c^ |  |  |
| Pharma industry pharmacovigilance  system | RDC n^o^ 4, article 3  Law n.º 6.360/76  article 79 |  |  | X^c^ |  |  |
| Importer/exported reporting of ADRs required | --- |  |  | X^c^ |  |  |
| Mandatory ADR reporting by health professionals | No |  |  | X^c^ |  |  |
| Stakeholder coordination |  |  |  |  |  |  |
| Pharmacovigilance system strengthening ^¶^ | --- | X^b^ | X^b^ | X^c^ |  |  |
| Regulatory Governance |  |  |  |  |  |  |
| Good Regulatory Practices Program | Ordinance  nº 422, April 2008 |  |  |  |  |  |
| Regulatory policy monitoring and evaluation | Bill 3.337/04  2004  (Regulatory Agenda) |  |  |  |  |  |
| Transparency^e^   - Supply chain management   (GGM) ^¶^   - Risk mitigation decisions ) | Decree nº 5.482,  June 2005  Complemen-tary Law nº 141 January 2012 | X^abd^ |  | X^c^ |  |  |
| Civil Society/public sector participation^[[4]](#endnote-4)^   - Public forums | Laws 8.080 Art. 7 VIII and 8.142, 1990  Ordinance 354 art.51  2006  Good regulatory practices program 2008 | X^d^ |  |  | X |  |
| Accountability   - Codes of ethics to prevent corruption - Regulatory impact analysis deployed as part of PMR | 2007 | X^d^ |  |  |  | X  2003^h^ |

Source:

^a^ Pal SN, Duncombe C, Dennis Falzon, Olsson S (2103) WHO Strategy for Collecting Safety Data in Public Health Programmes: Complementing Spontaneous Reporting Systems. *Drug Safety* 36, 75-81.

^b^UNAIDS | WHO. (2011). Technical Guidance Note for Global Fund HIV Proposals. From <http://www.unaids.org/en/media/unaids/contentassets/documents/programmes/programmeeffectivenessandcountrysupportdepartment/gfresourcekit/20110818_Technical_Guidance_Pharmacovigilance.pdf>

^c^PAHO. (2011). Buenas Prácticas de Farmacovigilancia *[Good pharmacovigilance practices for the Americas]* (pp. 78). Washington, D.C.: Organización Panamericana de la Salud.

^d^ Anello, E. (2008). A Framework for Good Governance in the Public Pharmaceutical Sector- Working draft for field testing and revision. Geneva: World Health Organization.

^e^ ANVISA at Página de Transparência Pública. Retrieved from <http://www3.transparencia.gov.br/TransparenciaPublica/index.jsp?Codi>

^h^ The OECD Expert Meeting on *Regulatory Performance: Ex post Evaluation of Regulatory Policies* ([OECD](#_ENREF_51)).

1. Endnotes

   Health professionals reporting: voluntary, Industry reporting: mandatory (Law 6360 art.79) [↑](#endnote-ref-1)
2. A prospective, observational, cohort study of adverse events associated with one or more medicines. [↑](#endnote-ref-2)
3. Healthcare professional screens for ADRs at each routine patient encounter. [↑](#endnote-ref-3)
4. Public consultation is pursuant to Ordinance 354/2006 art 51 [↑](#endnote-ref-4)
